# Knowledge of emergency management of avulsed tooth among Japanese dental students

**DOI:** 10.1186/1472-6831-14-34

**Published:** 2014-04-08

**Authors:** Yuko Fujita, Yasuhiro Shiono, Kenshi Maki

**Affiliations:** 1 Division of Developmental Stomatognathic Function Science, Department of Health Promotion, Kyushu Dental University, 2-6-1 Manazuru, Kokurakita-ku, Kitakyushu 803-8580, Japan

**Keywords:** Education, Dental trauma, Knowledge

## Abstract

**Background:**

The management of the avulsion of deciduous and permanent teeth in children is well outlined in the guidelines of the International Association of Dental Traumatology and the American Academy of Pediatric Dentistry. However, little information is available about the level of knowledge in the management of dental trauma among undergraduate dental students in Japan. The objective of this study is to explore dental students’ level of educational knowledge in the management of avulsed teeth.

**Methods:**

A three-part questionnaire was used to gather demographic data and evaluate the knowledge of students at Kyushu Dental University.

**Results:**

Questionnaire data were collected from 121 (53 first-year, 68 sixth-year) students. Regarding the immediate emergency management of a case in which a 9-year-old girl had fallen down the stairs and lost a maxillary incisor but remained conscious, 55.9% of sixth year students and 28.3% of first-year students suggested the immediate transportation of the tooth to a dentist. The answer selected by the largest number (50.9%) of first-year respondents was “sideline the injured girl and get her to bite on a tissue paper for several hours”. In a case in which a boy had an avulsed tooth after falling down on a road, only 13.2% of first-year students suggested the transportation of the tooth in his mouth to the clinic. Most the largest number of respondents believed that the best way to transport an avulsed tooth to the dental clinic was to “wrap it in dry tissue paper”.

**Conclusions:**

These results suggest that education in first aid for accidents that occur outside dental clinics or hospitals is insufficient. Japanese dentists and dental educations must immediately improve the utilization of the guidelines for dental trauma and the education of undergraduate students and patients in the management of dental trauma using an integrated approach.

## Background

The majority of dental injuries occur between the ages 8 and 11 years; falling accidents in a school environment are very common and are the main cause of dental trauma [1]. Tooth avulsion is the complete displacement of a tooth from its socket due to accidental or non-accidental injury; avulsion occurs in 1-16% of all dental injuries and may cause the loss of healthy teeth [1,2]. Avulsion is the most serious form of dental trauma. Many studies have investigated the knowledge of avulsed teeth in children among parents, school teachers, and general dentists, and have emphasized the necessity of education to prevent and improve the prognosis of avulsed teeth [3-8]. The management of the avulsion of deciduous and permanent teeth in children is well outlined in the guidelines of the International Association of Dental Traumatology [9] and the American Academy of Pediatric Dentistry [10]. However, only professional pediatric dentists are familiar with these guidelines in Japan, and general dentists hold diverse opinions about appropriate emergency procedures for injured teeth.

Japan’s Ministry of Education, Culture, Sports, Science and Technology reformed the dental education program and established a model core curriculum in March 2001. This curriculum has since been introduced in dental universities and has a primary goal of ensuring that undergraduate dental students can explain the clinical examinations, tests, diagnoses, treatments, and prognoses of dental trauma in children [11]. However, little information is available about the level of knowledge in the management of dental trauma among undergraduate dental students in Japan.

The aims of this study are to evaluate dental students’ level of educational knowledge in the management of avulsed teeth in children, and to investigate the influence of pediatric dental education on the management of dental trauma among undergraduate dental students.

## Methods

This study was approved by the Human Investigations Committee of Kyushu Dental University and all subjects provided written informed consent prior to participation.

This study used a modification of the questionnaire used by Al-Obaida [5] (Tables 1, 2, 3). Questionnaire data were collected from 121 students in two dental classes at Kyushu Dental University: 53 (32 men, 21 women) were first-year students and 68 (42 men, 26 women) were sixth-year students. The surveys were distributed in July 2011. First-year students entered the dental university after graduation from high schools in April 2011 and were attending general education courses: thus, they had little knowledge about dentistry at the time of this investigation. The lecture course about dental trauma in children and adolescents is in the fourth year of the curriculum at this university. The sixth-year students were attending clinical practice at the university hospital at the time of this investigation.

**Table 1:** Questionnaire of the personal information

Q1. Gender	1. Male
	2. Female
Q2. Age	1. 18~22
	2. 23~27
	3. 28~32
	4. 33~37
	5. >38
Q3. Did you have first-aid training of the dental trauma?	1. Yes
	2. No
Q4. Have you ever experienced an accident of dental trauma?	1. Yes
	2. No

**Table 2:** Questionnaire about the knowledge of dental injuries

**You are in a building at an elementary school. In front of you, a 9-year-old girl falls down the stairs and her lips receive a heavy blow. She is bleeding visibly from her mouth, and one upper front tooth is found to be missing. Fortunately, she did not lose consciousness.**	
Q5. Is the damaged front tooth likely to be a primary or permanent tooth?	1. A primary tooth
	2. A permanent tooth
Q6. Which of the following would you do? (Arrange in order of priority)	1. Wash the avulsed tooth with tap water.
	2. Put the avulsed tooth back into the socket immediately.
	3. Take her immediately to the nearest dentist with the avulsed tooth.
	4. Sideline the injured girl and get her to bite on a tissue paper for several hours to control the bleeding.
	5. Ask her whether she has incurred serious damage or injury.

**Table 3:** Questionnaire about medical knowledge of dental trauma

**A boy who lives in your neighborhood has fallen down on the road, and one of his teeth has fallen out. He came to you with the knocked-out tooth in his hand after the accident.**	
Q7. Would you replant (put back) the tooth into the socket from which it avulsed?	1. Yes
	2. No
Q8. If you decide to replant the tooth into its socket, but it has fallen onto the ground and is covered in dirt, what would you do?	1. Rinse the tooth under running water.
	2. Gently wipe off the mud that is stuck to the tooth by hand.
	3. Scrub the tooth gently with a toothbrush.
	4. Spray alcohol on the tooth.
	5. Put the tooth straight back into the socket, with no pretreatment.
Q9. If you did not replant the tooth, how would you transport it to the dentist?	1. Hold the tooth in a hand.
	2. Pack the tooth in ice.
	3. Seal the tooth in plastic wrap.
	4. Hold the tooth in the child’s mouth.
	5. Wrap the tooth in dry tissue paper.
Q10. If liquid is used to transport the tooth, how would you transport it to the dentist? (Arrange in order of priority)	1. Milk
	2. Tap water
	3. Alcohol
	4. Physiological saline
	5. Sports drink.

The questionnaire was divided into three parts. Part 1 consisted of questions about the personal profiles of the students (Table 1), part 2 contained questions about their knowledge of dental injuries (Table 2), and part 3 assessed the students’ medical knowledge of dental trauma (Table 3).

### Statistical methods

The data of the first-year students were compared with the data of the sixth-year students. Comparisons of frequencies of correct responses were analyzed with chi square test. The level of significance was set to 5%.

## Results

The students’ response rate was 64.0%. The students’ demographic characteristics are shown in Table 4. First-year students ranged in age from 18 to 27 years and sixth-year students were 23 to 41 years of age. One (1.9%) first-year student and all but six (91.2%) sixth-year students had first-aid training. Among all students, 20.7% had experience with at least one trauma case.

**Table 4:** Demographic characteristics of respondents to the questionnaire of the students

	**Responses (%)**	**Gender (%)**	**Age groups (%)**	**The experience of the training in dental emergencies (%)**	**The dental trauma experience (%)**
First-year students	N = 53/101 (52.5)	Male: 32 (60.4)	18 ~ 22: 52 (98.1)	Yes: 1 (1.9)	Yes: 14 (26.4)
		Female: 21 (39.6)	23 ~ 27: 1 (1.9)	No: 52 (98.1)	No: 39 (73.6)
			28 ~ 32: 0 (0)		
			33 ~ 37: 0 (0)		
			>38: 0 (0)		
Sixth-year students	N = 68/88 (77.3)	Male: 42 (61.8)	18 ~ 22: 0 (0)	Yes: 62 (91.2)	Yes: 11 (16.2)
		Female: 26 (38.2)	23 ~ 27: 56 (82.4)	No: 6 (8.8)	No: 57 (83.8)
			28 ~ 32: 7 (10.3)		
			33 ~ 37: 4 (5.9)		
			>38: 1 (1.5)		
Total	N = 121/189 (64.0)	Male: 74 (61.2)	18 ~ 22: 52 (43.0)	Yes: 63 (52.1)	Yes: 25 (20.7)
		Female: 47 (38.8)	23 ~ 27: 57 (47.1)	No: 58 (47.9)	No: 96 (79.3)
			28 ~ 32: 7 (5.8)		
			33 ~ 37: 4 (3.3)		
			>38: 1 (0.8)		

The first-year students answered an average of 2.66/6 questions correctly, whereas the sixth-year students answered an average of 4.79/6 questions correctly. The percentages of correct responses were significantly higher among sixth-year students than among first-year students for all questions except for question 10 (*P* < 0.025; Table 5).

**Table 5:** Correct responses to pediatric dental trauma questionnaire by dental students

	**1st Yr (%)**	**6th Yr (%)**	**Total (%)**	**p-value**
Q5				
Correct response: A permanent tooth	19 (35.8)	65 (95.6)	84 (69.4)	0.000
Q6				
Correct response: Take her immediately to the nearest dentist with the avulsed tooth.	15 (28.3)	38 (55.9)	53 (43.8)	0.002
Q7				
Correct response: Yes	24 (45.3)	49 (72.1)	73 (60.3)	0.003
Q8				
Correct response: Rinse the tooth under running water.	23 (43.4)	44 (64.7)	67 (55.4)	0.025
Q9				
Correct response: Hold the teeth in the child’s mouth.	7 (13.2)	65 (95.6)	72 (59.5)	0.000
Q10				0.556
Correct response: Fresh milk	14 (26.4)	39 (57.4)	53 (43.8)	
Physiological saline	23 (43.4)	29 (42.6)	52 (43.0)	

All data are analyzed by chi square tests.

**Figure 1:**
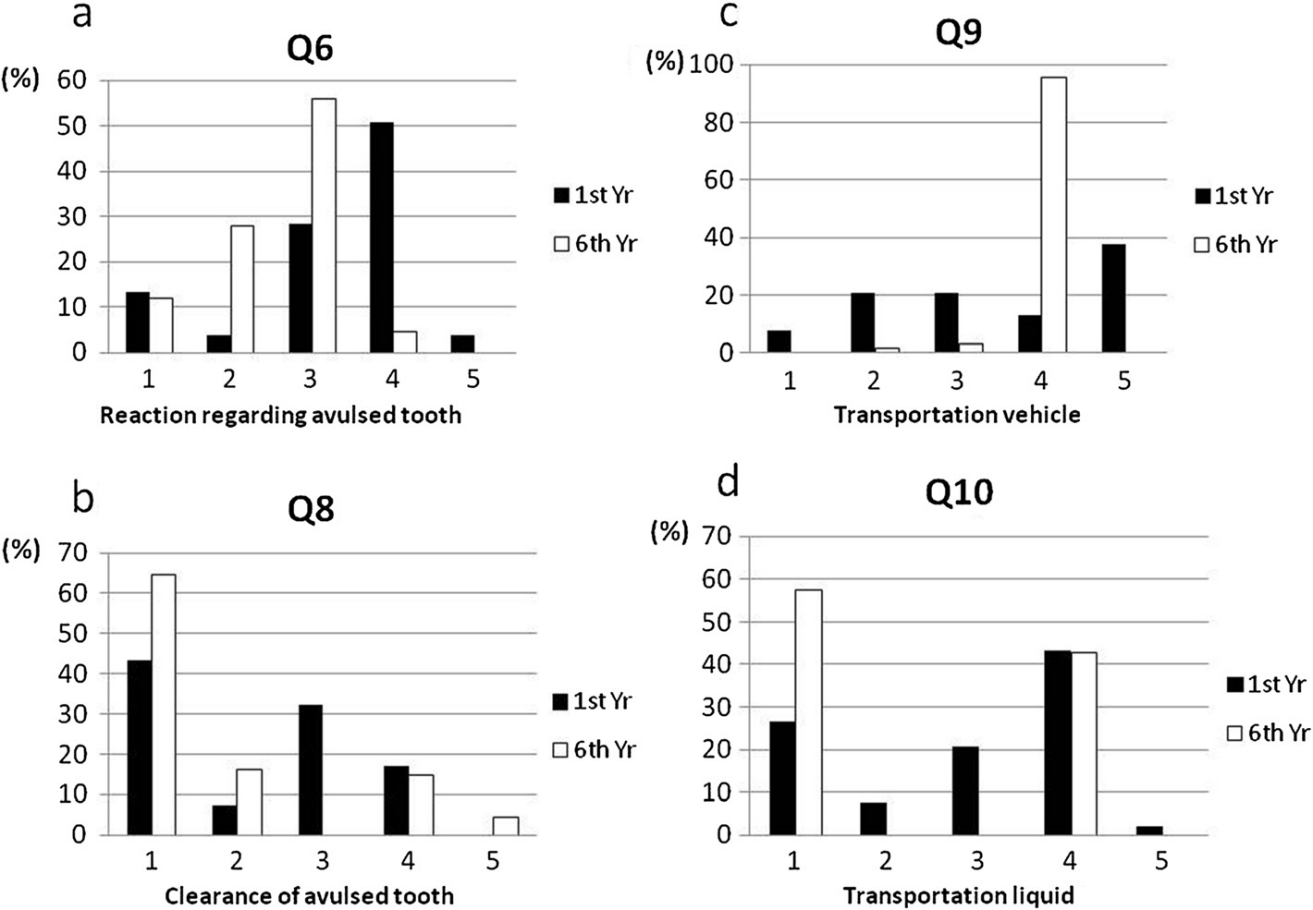
**Frequency distributions of dental students’ responses to the questions. (a)**. Dental students’ responses to question 6. 1. Wash the avulsed tooth with tap water. 2. Put the avulsed tooth back into the socket immediately. 3. Take her immediately to the nearest dentist with the avulsed tooth. 4. Sideline the injured girl and get her to bite on a tissue paper for several hours to control the bleeding. 5. Ask her whether she has incurred serious damage or injury. **(b)**. Dental students’ responses to question 8. 1. Rinse the tooth under tap water. 2. Gently wipe off the mud that is stuck to the tooth by hand. 3. Scrub the tooth gently with a toothbrush. 4. Spray alcohol on the tooth. 5. Put the tooth back into the socket immediately, with no pretreatment. **(c)**. Dental students’ responses to question 9. 1. Hold the tooth in a hand. 2. Pack the tooth in ice pickles. 3. Seal the tooth in plastic wrap. 4. Hold the tooth in the child’s mouth. 5. Wrap the tooth in dry tissue paper. **(d)**. Dental students’ responses to question 10. 1. Fresh milk. 2. Tap water. 3. Alcohol. 4. Physiological saline. 5. Sports drink.

The percentage of students who knew that the maxillary incisor of a 9-year-old girl is a permanent tooth was 35.8% among first-year students and 95.6% among sixth-year students. Regarding the immediate emergency management of a in which a 9-year-old girl fell down the stairs and lost a maxillary incisor but remained conscious, only 28.3% of first-year students gave the correct response. The answer selected by the largest number (50.9%) of respondents was “sideline the injured girl and get her to bite on a tissue paper for several hours to control the bleeding”. 55.9% of sixth-year students suggested the immediate transport of the tooth to a dentist (Table 5, Figure 1a).

For the scenario in which a boy had lost a tooth and held it in his hand after falling down on a road, 45.3% of first-year students and 72.1% of sixth-year students suggested the replantation of the avulsed tooth. Regarding the clearance of the dirty avulsed tooth, 43.4% of first-year students and 64.7% of sixth-year students gave the correct response (Table 5, Figure 1b).

Regarding the best way to transport the tooth to the dentist, 13.2% of first-year students and 95.6% of sixth-year students gave the correct response. The answer selected by the largest number (37.7%) of first-year respondents was to “wrap it in dry tissue paper” (Figure 1c). Regarding the best kind of liquid in which to transport the tooth to the dentist, all sixth-year students selected milk (57.4%) or physiological saline (42.6%). The answer selected by the largest number (43.4%) of first-year respondents was physiological saline (Table 5, Figure 1d).

## Discussion

This study found that only one (1.9%) first-year student had received first-aid training, suggesting that no such training for the management of dental emergencies had been provided in most elementary schools, junior high schools, and high schools. In Japan, faculty members specializing in pediatric dentistry provide lectures about dental trauma in the fourth year curricula of dental schools. The six sixth-year students who stated that they had received no training in the management of dental emergencies may have been absent from these lectures. These lectures follow the model core curriculum established by Japan’s Ministry of Education, Culture, Sports, Science and Technology [11]. In Europe, the *Profile and competences for the graduating European dentist – update 2009*[12] published by the Association for Dental Education in Europe (ADEE) states that a dentist must be competent in the management of trauma in the deciduous and permanent dentition upon graduation. The *Competences for the New General Dentists*[13] approved in 2008 by the House of Delegates of the American Dental Education Association (ADEA) states that graduates must be competent in the prevention, identification, and management of trauma, oral diseases, and other disorders. The educational policy in Japan is very similar to those presented in these European and American competencies.

In the present study, more than half of sixth-year students possessed basic knowledge of dental trauma in children. The percentages of correct responses were higher among sixth-year students than among first-year students for all questions. However, several insufficiencies were identified in the sixth-year students’ knowledge of dental trauma management. In particular, less than 70% of these students provided correct responses to questions 6 and 8. The guidelines for the management of dental trauma published by the International Association for Dental Traumatology (IADT) and the American Academy of Pediatric Dentistry (AAPD) recommend the immediate replantation of a tooth to obtain the best prognosis [9,10,14]. If the tooth cannot be replanted within 5 minutes, it should be stored in a medium that will help maintain the vitality of the periodontal ligament fibers [15]. In the immediate emergency management of such case, these two guidelines stipulate that the tooth should be washed with cold running water for a maximum of 10 seconds before replantation [9,10]. However, other studies have reported that such an approach is unsuccessful for many reasons, including the lack of knowledge about how to replant an avulsed tooth [16-18]. Thus, we believe that the immediate transportation of the tooth to a dentist, rather than replantation of the tooth, was a more appropriate choice in student responses.

IADT and AAPD guidelines for the management of dental trauma state that the physiological transportation media for avulsed teeth include Hank’s Balanced Salt Solution (tissue culture medium), saline, and cold milk [9,10,19-22]. In the present study, 43.4% of first-year students and 42.6% of sixth-year students selected physiological saline as the best transportation medium for avulsed teeth. In addition, 57.4% of sixth-year students selected milk, indicating that they know milk and saline are the most practical transport mediums for the storage of avulsed teeth because pH and osmolality of them are similar to those of extracellular fluid. On the other hand, recent study revealed that long shelf-life ultra-high temperature skim cow milk is not effective in preserving fibroblast viability *in vitro*[23]. Now, numerous kinds of milk are appearing on the markets in the world. Therefore, we need to educate the appropriate types of milk as the storage solutions of avulsed teeth to laypeople, dental students, and general dentists.

The first-year students were considered equivalent to people with no knowledge in the management of dental trauma. This study showed that less than 50% of these students provided correct answers to the five questions. These results are consistent with previous surveys of laypeople, who have been shown to the lack information about the prevention and management of dental trauma [24-26].

Regarding the immediate emergency management of the case involving the 9-year-old girl, the answer chosen by the largest number of respondents was to “sideline the injured girl and her to bite on a tissue paper for several hours to control the bleeding”. The students considered only the factors of bleeding or pain. Moreover, most the largest number of respondents believed that the best way to transport an avulsed tooth to the dental clinic was to “wrap it in dry tissue paper”, suggesting that they did not know that an avulsed tooth must not be dried because the risk of ankylosis increases significantly with an extraoral drying time of 20 minutes [10,15,27,28]. These results reflect the level of knowledge about dental emergencies among laypeople. We believe that dentists should take these findings into consideration and provide more instruction in the emergency management of the dental trauma to laypeople.

Rodd et al. [29] reported that previous studies in the United Kingdom have highlighted the lack of confidence and competence in trauma management among dentists, and dental students there have reported a lack of confidence in dental trauma management that warrants greater emphasis in the undergraduate curriculum. Vasconcellos et al. [30] also demonstrated that general dentists in Brazil need to improve their knowledge on avulsion and dental trauma prevention. Consistent with these studies, the results of the present study indicate that students nearing graduation must improve their knowledge level in dental trauma management.

The limitations of education in the management of dental trauma in Japan may be due to the lack of guidelines for the management of dental trauma and the diversity of opinions about appropriate management methods. Thus, the contents of lectures about dental trauma may be limited. Moreover, including instruction about clinical examinations, diagnoses, and treatment methods for dental trauma is important in Japan’s undergraduate curriculum. We believe that education in first aid for accidents that occur outside dental clinics or hospitals is insufficient. One must improve the knowledge level of undergraduate dental students in first-aid for dental trauma using educational methods such as problem-based learning or e-leaning.

## Conclusions

Japanese dentists and dental educations must immediately improve the utilization of the guidelines for dental trauma and the education of undergraduate students and patients in the management of dental trauma using an integrated approach. It will then be necessary for dentists to improve the knowledge of dental trauma management among laypeople.

## Competing interests

The authors declare that they have no competing interests.

## Authors’ contributions

YF formulated the study design, participated in data acquisition, analysis and drafted the whole manuscript. YS assisted in the analysis of the study. KM supervised the data analysis and interpretation, edited and gave the final approval of the manuscript. All authors read and approved the final manuscript.

## Pre-publication history

The pre-publication history for this paper can be accessed here:

http://www.biomedcentral.com/1472-6831/14/34/prepub

## References

[B1] Petersson EE Andersson L Sörensen S Traumatic oral vs non-oral injuries Swed Dent J 1997 21 55 68 9178450

[B2] Glendor U Marcenes W Andreasen JO Andreasen JO, Andreasen FM, Andersson L Classification, epidemiology and etiology Textbook and Color Atlas of Traumatic Injuries to the Teeth 2007 4 Oxford: Blackwell Munksgaard 217 254

[B3] Hegde AM Kumar KN Varghese E Knowledge of dental trauma among mothers in Mangalore Dent Traumatol 2010 26 417 421 10.1111/j.1600-9657.2010.00905.x 20636363

[B4] Oliveira TM Sakai VT Moretti AB Silva TC Santos CF Machado MA Knowledge and attitude of mothers with regards to emergency management of dental avulsion J Dent Child (Chic) 2007 74 200 202 18482514

[B5] Al-Obaida M Knowledge and management of traumatic dental injuries in a group of Saudi primary schools teachers Dent Traumatol 2010 26 338 341 10.1111/j.1600-9657.2010.00894.x 20662887

[B6] Abu-Dawoud M Al-Enezi B Andersson L Knowledge of emergency management of avulsed teeth among young physicians and dentists Dent Traumatol 2007 23 348 355 10.1111/j.1600-9657.2006.00477.x 17991234

[B7] Hamilton FA Hill FJ Holloway PJ An investigation of dento-alveolar trauma and its treatment in an adolescent population. Part 2: Dentists’ knowledge of management methods and their perceptions of barriers to providing care Br Dent J 1997 182 129 133 10.1038/sj.bdj.4809323 9061998

[B8] Kostopoulou MN Duggal MS A study into dentists’ knowledge of the treatment of traumatic injuries to young permanent incisors Int J Paediatr Dent 2005 15 10 19 15663440 10.1111/j.1365-263X.2005.00588.x

[B9] Andersson L Andreasen JO Day P Heithersay G Trope M Diangelis AJ Kenny DJ Sigurdsson A Bourguignon C Flores MT Hicks ML Lenzi AR Malmgren B Moule AJ Tsukiboshi M International Association of Dental Traumatology guidelines for the management of traumatic dental injuries: 2. Avulsion of permanent teeth Dent Traumatol 2012 28 88 96 10.1111/j.1600-9657.2012.01125.x 22409417

[B10] American academy of pediatric dentistry Guidelines for the management of traumatic dental injuries: 2. Avulsion of permanent teeth [http://www.aapd.org/media/Policies_Guidelines/E_Avulsion.pdf] 27931479

[B11] The Ministry of Education, Culture, Sports, Science and Technology of Japan [http://www.mext.go.jp/b_menu/shingi/chousa/koutou/033-1/toushin/1304433.htm]

[B12] Cowpe J Plasschaert A Harzer W Vinkka-Puhakka H Walmsley AD Profile and competences for the graduating European dentist - update 2009 Eur J Dent Educ 2010 14 193 202 10.1111/j.1600-0579.2009.00609.x 20946246

[B13] Competences for the New General Dentists as approved by the 2008 American Dental Education association (ADEA) House of Delegates J Dent Educ 2011 75 932 935

[B14] Trope M Clinical management of the avulsed tooth: present strategies and future directions Dent Traumatol 2002 18 1 11 10.1046/j.1600-4469.2001.00001.x 11841460

[B15] Sigalas E Regan JD Kramer PR Witherspoon DE Opperman LA Survival of human periodontal ligament cells in media proposed for transport of avulsed teeth Dent Traumatol 2004 20 21 28 10.1111/j.1600-4469.2004.00219.x 14998411

[B16] Chan AW Wong TK Cheung GS Lay knowledge of physical education teachers about the emergency management of dental trauma in Hong Kong Dent Traumatol 2001 17 77 85 10.1034/j.1600-9657.2001.017002077.x 11475950

[B17] Holan G Shmueli Y Knowledge of physicians in hospital emergency rooms in Israel on their role in cases of avulsion of permanent incisors Int J Paediatr Dent 2003 13 13 19 10.1046/j.1365-263X.2003.00414.x 12542619

[B18] Glendor U Has the education of professional caregivers and lay people in dental trauma care failed? Dent Traumatol 2009 25 12 18 10.1111/j.1600-9657.2008.00707.x 19208006

[B19] Barrett EJ Kenny DJ Survival of avulsed permanent maxillary incisors in children following delayed replantation Endod Dent Traumatol 1997 13 269 275 10.1111/j.1600-9657.1997.tb00054.x 9558508

[B20] Andreasen JO Borum MK Jacobsen HL Andreasen FM Replantation of 400 avulsed permanent incisors. 4. Factors related to periodontal ligament healing Endod Dent Traumatol 1995 11 76 89 10.1111/j.1600-9657.1995.tb00464.x 7641622

[B21] Barrett EJ Kenny DJ Avulsed permanent teeth: a review of the literature and treatment guidelines Endod Dent Traumatol 1997 13 153 163 10.1111/j.1600-9657.1997.tb00031.x 9550040

[B22] Hiltz J Trope M Vitality of human lip fibroblasts in milk, Hanks balanced salt solution and Viaspan storage media Endod Dent Traumatol 1991 7 69 72 10.1111/j.1600-9657.1991.tb00187.x 1782897

[B23] Moura CC Soares PB de Paula Reis MV Fernandes Neto AJ Zanetta Barbosa D Soares CJ Potential of coconut water and soy milk for use as storage media to preserve the viability of periodontal ligament cells: an in vitro study Dent Traumatol 2014 30 22 26 10.1111/edt.12042 23566116

[B24] Traebert J Traiano ML Armênio R Barbieri DB de Lacerda JT Marcenes W Knowledge of lay people and dentists in emergency management of dental trauma Dent Traumatol 2009 25 277 283 10.1111/j.1600-9657.2009.00779.x 19583575

[B25] Qazi SR Nasir KS First-aid knowledge about tooth avulsion among dentists, doctors and lay people Dent Traumatol 2009 25 295 259 10.1111/j.1600-9657.2009.00782.x 19583578

[B26] Díaz J Bustos L Herrera S Sepulveda J Knowledge of the management of paediatric dental traumas by non-dental professionals in emergency rooms in South Araucanía, Temuco, Chile Dent Traumatol 2009 25 611 619 10.1111/j.1600-9657.2009.00835.x 19843130

[B27] Chappuis V von Arx T Replantation of 45 avulsed permanent teeth: a 1-year follow-up study Dent Traumatol 2005 21 289 296 10.1111/j.1600-9657.2005.00330.x 16149925

[B28] Donaldson M Kinirons MJ Factors affecting the time of onset of resorption in avulsed and replanted incisor teeth in children Dent Traumatol 2001 17 205 209 11678538 10.1034/j.1600-9657.2001.170503.x

[B29] Rodd HD Farman M Albadri S Mackie IC Undergraduate experience and self-assessed confidence in paediatric dentistry: comparison of three UK dental schools Br Dent J 2010 208 221 225 10.1038/sj.bdj.2010.207 20228757

[B30] de Vasconcellos LG Brentel AS Vanderlei AD de Vasconcellos LM Valera MC de Araújo MA Knowledge of general dentists in the current guidelines for emergency treatment of avulsed teeth and dental trauma prevention Dent Traumatol 2009 25 578 583 10.1111/j.1600-9657.2009.00820.x 19788428

